# Probiotic Fermented Goat’s and Sheep’s Milk: Effect of Type and Dose of Collagen on Survival of Four Strains of Probiotic Bacteria during Simulated In Vitro Digestion Conditions

**DOI:** 10.3390/nu15143241

**Published:** 2023-07-21

**Authors:** Kamil Szopa, Katarzyna Szajnar, Małgorzata Pawlos, Agata Znamirowska-Piotrowska

**Affiliations:** Department of Dairy Technology, Institute of Food Technology and Nutrition, University of Rzeszow, Ćwiklińskiej 2D, 35601 Rzeszow, Poland; kszajnar@ur.edu.pl (K.S.); mpawlos@ur.edu.pl (M.P.)

**Keywords:** probiotic, bacterial survival rate, in vitro, caprine milk, ovine milk, fermented goat’s milk, fermented sheep’s milk, bovine collagen, collagen hydrolysate

## Abstract

Microbial tolerance of digestive stresses depends not only on the bacterial strain but also on the structure and physicochemical properties of the supply chain and the foods that contain it. In the present study, we aimed to evaluate the effects of the type of milk (ovine, caprine) and the type and dose of collagen on the viability of four probiotic strains, *Lacticaseibacillus paracasei* L-26, *Lacticaseibacillus casei* 431, *Lactobacillus acidophilus* LA-5, and *Lacticaseibacillus rhamnosus* Lr-32, during in vitro gastrointestinal digestion. The highest survival rate under simulated in vitro digestion conditions compared to the number of cells before digestion was found in two strains, *L. casei* and *L. paracasei*, where survival rates were greater than 50% in each batch. The survival rate of the *L. rhamnosus* strain ranged from 41.05% to 64.23%. In caprine milk fermented by *L. acidophilus*, a higher survival rate was found in milk with 1.5% hydrolysate than the control, by about 6%. Survival of the *L. rhamnosus* strain was favorably affected by the 3% addition of bovine collagen in caprine milk, which increased survival by about 14% compared to the control sample. Adding 3% of hydrolysate to sheep’s and goat’s milk enhanced the survival of the *L. rhamnosus* strain by 3% and 19%, respectively. This study reports that fermented caprine and ovine milk may be suitable matrices for the probiotic supply of commercial dairy starter cultures and promote gut homeostasis.

## 1. Introduction

Dairy products, including yoghurt, fermented sour milk, and cheese, currently remain at the forefront of probiotic food development [1]. Profound attention is being paid to products enriched with probiotic bacteria due to the well-known benefits of consuming sufficient amounts of these microorganisms [2,3]. According to statistical data, from 2015 to 2025, the global market for probiotic supplements is expected to expand from USD 3.3 billion to USD 7.0 billion [4].

Numerous widely consumed and currently commercially available probiotic strains have GRAS safety status in the U.S. or belong to species with a qualified presumption of safety (QPS) status from the European Food Safety Authority (EFSA) [1,5]. Probiotic effects are strain-specific. These organisms are credited with numerous health benefits, such as anti-infective, antimutagenic, and anticancer, and could be used as an alternative to antiviral therapy and for lowering cholesterol, improving nutritional value, and alleviating symptoms of lactose intolerance [6,7,8,9,10]. One of the mechanisms by which probiotics improve health is stimulating the systemic immune system [11,12]. Lactic acid bacteria bind to particular receptors on immune cells and other organs, such as the intestinal epithelium, and initiate the production of cytokines, chemokines, and T and B lymphocytes, activating the D.C. dendritic cells and macrophages [13]. By secreting metabolites, including bacteriocins, organic acids, short-chain fatty acids, and hydrogen peroxide, probiotics may also activate the mucosal immune system. The metabolites exhibit antimicrobial properties, preventing the proliferation of selected pathogens. Bacteriocins affect bacterial cytoplasmic membranes and target stimulated membrane vesicles to disrupt proton-motive force, thereby inhibiting the growth of many pathogens [10,14,15,16]. A key aspect of host colonization is the bacteria’s ability of adhesion, allowing it to adhere to other cells or surfaces. It represents an essential process for the survival and proliferation of probiotic bacteria in the gastrointestinal tract [17]. The ability to adhere is considered an essential prerequisite for colonization, inhibition of the growth of pathogenic microflora, immune interactions, and strengthening of intestinal barrier function. The ease of adhesion to the mucosal surface of the intestinal epithelium is one of the most important criteria for selecting probiotic bacteria [18]. The anticancer effect of probiotics is based on inhibiting intestinal bacterial enzymes that convert procarcinogens into carcinogens [19]. Certain strains of probiotic bacteria have been shown to effectively bind to and neutralize aflatoxin B1 (AFB1), thereby reducing the bioabsorption of the toxin from the gut. In addition, they decrease the levels of fecal enzymes such as glycosidase, B-glucuronidase, azoreductase, and nitroreductase, as well as secondary bile acid salts, and they reduce the absorption of harmful mutagens that lead to colon cancer [16,19].

Probiotic cultures should be resistant to the conditions of the gastrointestinal tract, particularly its acids, and should also be able to adhere to human intestinal epithelial cells, providing antimicrobial protection against harmful bacteria [20,21]. Probiotics must be provided in the food product in sufficient amounts before expiration, pass through the gastrointestinal tract, and colonize the gut in the number required for a measurable beneficial effect [22,23]. Due to low gastric pH, most probiotics cannot survive at high levels, which limits their effectiveness when producing functional foods. Resistance to gastric acid and enzymes, and tolerance of bile acid salts, are properties of microorganisms that allow them to survive in the acidic conditions of the stomach and survive the presence of bile acid salts in the small intestine during passage through the digestive system [24,25,26,27]. According to Okpara et al. [28], probiotics should be present in food at 10^8^–10^9^ cfu g^−1^ to ensure a sufficient therapeutic minimum once they reach the colon, i.e., 10^6^–10^7^ cfu g^−1^ [28]. The fact is that, during food processing and storage, microbial cells meet several different stresses that critically affect their viability [29,30]. The microorganism carrier is also essential. It has been found that a milk-based matrix is probably the best probiotic carrier in terms of maintaining a sufficient number of viable bacteria to sustain therapeutic effects in humans [31].

One of the problems concerning probiotic strains is the survival of microorganisms in the gastrointestinal tract. The effect of gastric acid, mainly responsible for neutralizing potentially pathogenic microorganisms ingested with food, is the primary barrier to probiotic bacteria [18]. Milk and dairy products that naturally contain lactic acid bacteria, including probiotic bacteria, are generally a good medium for their viability during storage [22,32]. The survival of probiotics in the gastrointestinal tract is favored by fat globules, which can protect viable cells from the acidic conditions of the stomach and bile salts [22]. Among dairy products of non-cow origin, caprine and ovine milk are the main carriers of probiotics [31]. Milk, due to its suitable properties such as proper pH, good buffering capacity, and high nutrient content, could improve the survival rate of probiotics. Moreover, milk’s fat and solid content could protect probiotic bacteria passing through the digestive tract [31]. Adding amino acids in the form of collagen and the presence of milk proteins could stimulate the growth of probiotic bacteria and their survival [32].

Recently, there has been an increasing demand for non-cow’s milk and milk products. Each type other than bovine milk is unique and has specific characteristics, such as nutritional value, physicochemical properties, and proposed therapeutic properties, making them potentially beneficial in human medicine and nutrition [31,33,34]. 

Caprine milk is an excellent source of fatty acids, protein, and minerals. Compared to bovine milk, it is characterized by a lower content of volatile fatty acids, a higher content of medium- and short-chain fatty acids, a lower proportion of casein micelles of the α_S1_-casein fraction, a smaller size of casein micelles, and a higher content of calcium and inorganic phosphorus [35,36]. Milk fat composition and structure are the main differences between bovine and caprine milk [33]. The average size of goat’s milk fat globules is smaller than cow’s milk [33]. Caprine milk has similar levels of vitamins C and D compared to the milk of bovine origin, and it has higher amounts of vitamins A and B. It is an abundant source of riboflavin (B2) and niacin (B3), which are essential in child growth [36]. The importance of caprine milk as a functional food is due to its high digestibility and nutritional value, as well as its medicinal and dietary properties, and it is, therefore, an excellent substitute for bovine milk in the nutrition of children and the elderly [35,37,38].

Ovine milk has also been identified as a suitable matrix for probiotic delivery and is promising for developing functional dairy products [20]. Sheep’s milk processing produces foods with an interesting nutritional profile and good yields (higher dry matter content) compared to milk from other domestic mammals [20]. Due to its favorable properties, it can serve as a valuable raw material for producing fermented beverages, which are the basis of the functional food market. Ovine milk differs from bovine milk in chemical composition and physicochemical properties. It is characterized by a high content of dry matter, fat, total protein, vitamins B, C, A, and D, as well as a high content of calcium, phosphorus, potassium, sodium, chlorine, and selenium, compared to bovine milk [39]. It is considered a source of functional bioactive peptides involved in processes related to the functioning of the digestive, endocrine, immune, and nervous systems [40]. Ovine milk products have therapeutic effects and are of great interest to consumers, especially people with limited lactose tolerance [41].

One of the difficulties of using probiotic bacteria is maintaining the viability of these cultures. In addition to the documented health-promoting properties of a particular strain, justifying the health benefits of using probiotic cultures requires evidence of the bacteria’s survival in the human gastrointestinal tract and its ability to multiply [42]. Studies are being conducted to assess the survival of microorganisms in gastrointestinal fluids by modelling the digestion process using in vitro tests [28,42,43]. In vitro gastrointestinal models are widely used to study survival and bioavailability by simulating gastrointestinal conditions [44]. It is known that the tolerance of microorganisms to digestive stresses depends not only on the microbial strain but also on the structure and physicochemical properties of the delivery system, as well as of the food that contains them [45,46]. This study aimed to evaluate the effect of the type of milk (sheep’s, goat’s) and the type and dose of collagen on the viability of four strains of probiotic bacteria, *Lacticaseibacillus casei*, *Lactobacillus acidophilus*, *Lacticaseibacillus paracasei*, and *Lacticaseibacillus rhamnosus*, at each phase (oral, stomach, small intestine) of simulated in vitro digestion conditions.

## 2. Materials and Methods

### 2.1. Materials

Raw ovine milk was collected in May from farms in the area of Nowy Sącz and Zakopane in Lesser Poland Province (Poland), while caprine milk was obtained in June from a farm in Subcarpathia Province (Zabratówka, Poland), for the manufacture of probiotic fermented milk. 

Two different types of collagen were used: 100% collagen protein hydrolysate (Vitagel-Collagen, Superior, Dobre Miasto, Poland) and 100% bovine collagen (FH Kol-Pol, Dębica, Poland). Four strains of not genetically modified dairy starter cultures were used for milk fermentation: *Lactobacillus acidophilus* LA-5^®^, *Lacticaseibacillus casei* 431^®^ (Chr. Hansen, Hoersholm, Denmark), *Lacticaseibacillus paracasei* L-26 (DELVO^®^ PRO, DSM, Moorebank, Australia), and *Lacticaseibacillus rhamnosus* Lr-32^®^ (Danisco, DuPont, Copenhagen, Denmark).

For the in vitro digestion enzymes and reagents used, thermostable α-amylase (TDF-100A; 24,975 U/mL), porcine stomach mucin (type II), pepsin from porcine gastric mucosa (250 U/mg solids), porcine bile extract, and pancreatin from porcine pancreas (8×USP specifications) were purchased from Sigma-Aldrich (St. Louis, MO, USA); and anhydrous disodium hydrogen phosphate ≥99.0% (Na_2_HPO_4_; 141.96 g/mol), dipotassium hydrogen phosphate (K_2_HPO_4_; 174.18 g/mol), sodium chloride ≥99.9% (NaCl; 58.44 g/mol), 12 mol hydrochloric acid (HCl), and 1 mol sodium hydroxide (NaOH) were provided by Chempur (Piekary Śląskie, Poland).

### 2.2. Fermented Milk Manufacture

Raw ovine and caprine milk were heated at 85 °C for 30 min [47,48,49]. The cooled milk was divided into a total of 40 batches (20 batches for sheep’s milk and 20 batches for goat’s milk) based on the added probiotic strains (*L. casei*, *L. acidophilus*, *L. paracasei*, and *L. rhamnosus*) and different forms of collagen (bovine and protein hydrolysate) and doses (1.5% and 3.0%).

The milk was divided into batches (Table 1 and Table 2). For each probiotic strain, ten batches of mixtures with additives were prepared [48,49]:

1. Sheep’s milk.
2. Sheep’s milk + 1.5% bovine collagen.
3. Sheep’s milk + 3.0% bovine collagen.
4. Sheep’s milk + 1.5% collagen protein hydrolysate.
5. Sheep’s milk + 3.0% collagen protein hydrolysate.
6. Goat’s milk.
7. Goat’s milk + 1.5% bovine collagen.
8. Goat’s milk + 3.0% bovine collagen.
9. Goat’s milk + 1.5% collagen protein hydrolysate.
10. Goat’s milk + 3.0% collagen protein hydrolysate.

The collagen–milk mixtures were heated to 60 °C, homogenized (20 MPa; CAT UNIDRIVE X 1000 D, Ballrechten-Dottingen, Germany) and re-pasteurized (85 °C, 10 min) according to Ramasubramanian et al. [50] and the directives of Commission Regulation (EC) No. 1662/2006 [51]. Next, the collagen–milk mixtures were cooled down to a temperature of 37 ± 1 °C. Then, 5% of one of the four pre-activated probiotic monocultures was added to five various batches of mixtures. The inoculum was obtained according to Szajnar et al. [52]. The bacterial inoculum contained about 9 log cfu g^−1^ of bacteria. A total of 40 batches of collagen–milk mixtures were obtained [48,49]. Each sample was stirred, transferred to 100 mL plastic cups, and fermented at 37 ± 1 °C until reaching a pH = 4.6 ± 0.2 (12–15 h). Next, the fermented-milk–collagen samples were cooled to 5 °C (ILW 115 Refrigerated Incubator, POL-EKO, Wodzisław Śląski, Poland) and cold-stored for five days. The experiment was repeated three times.

### 2.3. In Vitro Digestion Process

In vitro gastrointestinal digestion was carried out using a slightly modified method according to Buniowska et al. [53] and Silva et al. [54]. Simulated in vitro digestion of fermented milk was conducted after five days of cold storage at 5 °C. The three-phase in vitro digestion model included digestion phases in the oral cavity, stomach, and small intestine. 

Phase I, oral cavity: 50 g of each probiotic fermented milk was transferred to an Erlenmeyer flask. Then, 5 mL of saliva solution was added (a solution for 1 L of distilled water: 2.38 g of Na_2_HPO_4_, 0.19 g of K_2_HPO_4_, 8 g of NaCl, 100 mg/L mucin, and 150 mg/L α-amylase with an enzymatic activity of 200 U/L). Next, with 12 M HCl and 1 M NaOH, the pH of the obtained mixture of fermented milk and saliva was adjusted to 6.75 ± 0.2. The oral contents were incubated at 37 °C in a laboratory shaker (90 RPM, 10 min).

Phase II, stomach: 13.08 mg pepsin was added to the oral contents. The pH value was decreased to 2.0 ± 0.20 by adding HCl (12 mol/L). The sample was then incubated in a laboratory shaker at 37 °C and 90 RPM for two hours. 

Phase III, small intestine: 5 mL pancreatin (4 g/L) and bile salt (25 g/L) were added to the contents after the stomach phase at pH 7.00 ± 0.20 (HCl 12 mol/L or NaOH 1 mol /L). The mixture was placed in a laboratory shaker at 37 °C (90 RPM, 2 h). 

### 2.4. Microbiological Analysis

The viable counts of the four probiotic strains before and after each phase of gastrointestinal digestion were evaluated using the plate method on MRS agar as described by Znamirowska et al. [55] and Lima et al. [56]. Incubation was carried out in a vacuum desiccator with a GENbox aerator (Biomerieux, Warsaw, Poland) under anaerobic conditions (37 °C, 72 h). Grown colonies were counted with a colony counter (TYP J-3, Chemland, Stargard Szczecinski, Poland). The results were presented as log cfu g^−1^. 

The survival rate (%) was calculated by using the number of viable colonies of probiotic bacteria remaining in the intestinal contents compared to the undigested sample, according to equation [16]:

$$\mathrm{Survival}\;\mathrm{rate}\;\mathrm{of}\;\mathrm{probiotic}\;\mathrm{bacteria}\;(\%)=\frac{\mathrm{Viable}\;\mathrm{counts}\;\mathrm{of}\;\mathrm{probiotic}\;\mathrm{bacteria}\;\mathrm{in}\;\mathrm{digested}\;\mathrm{sample}}{\mathrm{Viable}\;\mathrm{counts}\;\mathrm{of}\;\mathrm{probiotic}\;\mathrm{bacteria}\;\mathrm{in}\;\mathrm{non}\;-\;\mathrm{digested}\;\mathrm{sample}}\;\times \;100$$

### 2.5. Statistical Analysis

From the obtained results, the mean and standard deviation were calculated using Statistica v. 13.1 software (StatSoft, Tulsa, OK, USA). One-way, two-way, and three-way ANOVA analysis was carried out. Tukey’s test (*p* ≤ 0.05) was applied to confirm the significance of differences between mean values.

## 3. Results and Discussion

The number of bacterial cells in fermented milk before digestion was determined to evaluate the effect of type and dose of collagen on the viability of probiotic strains during the gastrointestinal passage. The results of the live cell counts of the probiotic bacteria *L. acidophilus*, *L. casei*, *L. paracasei*, and *L. rhamnosus* are shown in Table 3, Table 4, Table 5 and Table 6. The analysis of the number of live bacterial cells before digestion indicated that ovine fermented milk showed more viable cells by 0.2–1.9 log cfu g^−1^ than caprine milk, considering similar fermentation conditions. Numerous studies confirmed that ovine milk contains higher dry matter, total protein, crude fat, casein, minerals, and vitamins compared to caprine milk [39,40,48,49].

Before digestion, the most significant differences between ovine and caprine milk were found for milk fermented by *L. paracasei* and *L. rhamnosus*. Moreover, a significantly lower number of bacterial cells was determined in sheep’s milk fermented by *L. paracasei* with the addition of 3% hydrolysate compared to control milk. However, before digestion, ovine milk fermented by *L. rhamnosus* with 1.5% and 3% hydrolysate demonstrated a significantly higher number of viable bacterial cells than the control. In comparison, goat’s milk with bovine collagen and hydrolysate showed a lower number of cells of the *L. rhamnosus* strain compared to the control milk. Sun et al. [57] confirmed that studying the composition of nutrient solutions for *L. rhamnosus* shows that the bacteria have specific requirements for both the type and ratio of amino acids in the nutrient solutions. Moreover, vitamin B2 (riboflavin) is required for the growth of *L. rhamnosus*, and caprine milk contains only 0.13 mg of it in 100 g, while in ovine milk, riboflavin is 0.36 mg in 100 g, which could be the reason for poorer growth [57,58,59,60].

The results shown in Table 3, Table 4, Table 5 and Table 6 indicated no significant reduction in probiotic viability in ovine milk fermented by the four probiotic strains at the end of the oral phase compared to the results before digestion. Also, studies by Kowalczyk et al. [16] and Melchior et al. [27] showed no significant effect of saliva activity on the viability of probiotic bacteria. This is related to the pH of saliva (6.5–7.0), which is optimal for the probiotics used and, in this environment, is suitable for the survival of bacterial cells [61]. Moreover, food degradation is progressive due to the action of the α-amylase enzyme present in saliva. Fermented milk is a low-viscosity, low-fluidity food with a relatively short exposure time to the enzyme in saliva [62]. After the oral phase, only a slight reduction in viability was observed in all samples, probably because this phase is characterized by a short exposure (2 min) at neutral pH without antimicrobial substance [2].

Low stomach acidity is the first barrier to microorganisms. As is commonly known, the pH in the stomach before eating a meal is different compared to during a meal. Before ingestion, the pH of the stomach is about 2.0. After ingestion, the pH increases to pH 5.5–7.0 within 2–3 h, while 1 h after the meal, the pH decreases to 2.5–4.0 [62,63]. In our study, the pH was lowered to about 2.0 at the stomach phase. During passage through the stomach, there was a reduction in the bacterial population in control ovine milk (OLP, OLR, OLC, OLA) by 4.46 log cfu g^−1^ in OLA milk with *L. acidophilus* and by 6.15 log cfu g^−1^ in OLR milk with *L. rhamnosus* compared to the number of cells in milk before digestion (Table 3, Table 4, Table 5 and Table 6). Similarly, the number of viable cells in caprine milk (KLP, KLR, KLC, KLA) in the stomach phase decreased by 3.50 log cfu g^−1^ in KLA milk with *L. acidophilus* and by 5.21 log cfu g^−1^ in KLR milk with *L. rhamnosus* relative to the number of bacterial cells before digestion. It is important to note that the milk type significantly influences the bacteria’s survival in the stomach phase. The survival rate of probiotic bacteria in caprine milk in this example was 3–9% higher than in ovine milk, except for the *L. casei* strain, for which it was the opposite. Nguyen et al. [64] found that almost all the proteins in ovine yoghurt are degraded most intensively in the human stomach during the first phase of digestion, in contrast to bovine and caprine yoghurt. In rodent studies conducted by Dalziel et al. [65], consumption of caprine milk resulted in faster gastric emptying compared to bovine milk, which was most likely related to the different coagulation properties of the two types of milk. Faster stomach emptying resulted in shorter exposure to adverse pH conditions [66]. In contrast, a study by Ziarno and Zaręba [67] showed that increasing dry matter by adding milk proteins and a higher milk fat content (2% or more) had a statistically significant beneficial effect on bacterial cell survival under simulated conditions. Meanwhile, in our study, only the *L. casei* strain showed this beneficial effect since sheep’s milk had a higher dry matter content of about 8% and fat content of about 6%. Also, it should be noted that, at the phase of digestion in the stomach, the beneficial effect of the addition of collagen on the survival rate of this strain was demonstrated.

The survival rates of the other strains under gastric conditions were variable (Table 3, Table 4, Table 5 and Table 6). The least resistant to gastric acid was the *L. rhamnosus* strain, with survival rates ranging from 35.20% in OLR1.5W to 46.67% in KLR3.0H compared to their counterparts before digestion. The best survival rate of 64.93% at the stomach phase was found in caprine milk KLA1.5H with hydrolysate and *L. acidophilus* compared to the results before the digestion. The addition of collagen at the stomach phase only favorably affected the survival rate of *L. casei* and *L. paracasei*, increasing it by 2–9%. In contrast, the dose of collagen had no significant effect on the number of probiotic cells in the gastric phase.

The ability of bacterial cells to tolerate acidic conditions is attributed to their capacity to maintain a constant pH gradient between the environment and the pH of the cytoplasm [67]. This is related to the enzyme F0F1-ATPaze, which, when induced at low pH, may increase intracellular pH at low extracellular pH, thereby protecting Gram-positive bacteria from an acidic environment [16]. This may explain the variability in results published in the literature, depending on the dose of the additive and the strain being studied for survival. The tolerance of lactic bacteria to an acidic environment depends on the enzymatic profile and composition of the cytoplasmic membrane, which depends on the type of bacteria and external conditions [68]. Probiotics must survive in the stomach’s acidic environment to reach the small intestine and recolonize the host. It is believed that *Lactobacillus* species are inherently resistant to acid, and acid resistance is one of the selection criteria for probiotics [16]. The optimal temperature and pH for the growth of lactic acid bacteria are 30–40 °C and pH 5.5–6.2, respectively, and are variable depending on the strain. Some strains show the ability to grow at temperatures ranging from 2 °C to 53 °C and pH ranging from 4.5 to 7.0 due to their potential to regulate intracellular pH. *L. casei* and *L. acidophilus* can grow at a pH below 4.4 [69,70,71]. For *L. rhamnosus*, the optimal pH value is 6.4 to 6.9, and the minimum is 4.4 to 3.4 [52]. *L. paracasei* survives exceptionally well in a strongly acidic environment with pH = 2.5 [72]. Strongly acidic conditions typical of the stomach environment can damage cell membranes of DNA and proteins [16,27,73]. Ospanov et al. [22] also observed a decrease in the number of bacterial cells during gastric passage. A study by Afzaal et al. [74] also confirmed that low pH (2.0) contributed to a reduction in the number of *L. casei* in simulated gastric juice. A study by Galdino et al. [68] reported a 2.03–2.68 log decrease in *L. rhamnosus* in fermented caprine milk after the stomach phase. In a study by Melchior et al. [27], the application of whey protein improved the survival of *L. rhamnosus* bacteria during in vitro digestion. The viability of probiotics is preserved despite the possible destructive activity of proteolytic enzymes during the gastric and intestinal phases due to the buffering capacity exerted by whey proteins [27]. A similar protective effect of whey protein isolate application on *L. rhamnosus* was demonstrated by Doherty et al. [75]. However, unfortunately, our study did not confirm the beneficial effect of collagen, a protein, on the survival of *L. rhamnosus* in the stomach phase. Moreover, the exopolysaccharides (EPSs) produced by probiotic bacteria show protective properties against extreme acidity or contact with bile salts [62]. Our study found the lowest survival rate during stomach and intestinal digestion in milk fermented by *L. rhamnosus*. This could be related to the fact that these bacteria produce low concentrations of exopolysaccharides compared to the other strains used.

Population reduction between the stomach and intestinal phases occurs due to temporary stress caused by pH changes in both phases. The low pH of the stomach phase causes damage to microbial cells, which can be rebuilt in the intestinal phase [68]. Food components are an essential protective factor for bacterial cells as components of the buffer food environment where the bacteria are suspended [67]. In the intestinal digestion phase, bile acid salts significantly impact bacterial survival. In the intestinal phase, where bile acid salts are responsible for protein and DNA damage and the emulsification of fats and the bacterial lipid membrane, whey proteins can form a barrier that reduces bile damage to lipid membranes [27].

Our studies indicated that the behavior of probiotic strains is very different after exposure to a simulated intestinal environment in vitro. In the intestinal phase in ovine milk fermented by *L. paracasei*, there were no significant differences due to the addition of collagen and hydrolysate compared to the stomach phase (Table 3). However, in caprine milk, adding hydrolysate (KLP1.5H, KLP3.0H) significantly increased the number of viable *L. paracasei* cells by 0.5 log cfu g^−1^ compared to the number of cells identified in the stomach.

At the intestinal phase in sheep’s milk fermented by *L. casei* (Table 5), there was a tendency for the population to increase in OLC control milk, while in milk with collagen and hydrolysate, there were no significant differences in the number of viable cells compared to the stomach phase. However, in goat’s milk, adding hydrolysate (KLC1.5H, KLC3.0H) reduced the *L. casei* population by 0.5–0.7 log cfu g^−1^ compared to the stomach phase.

The findings shown in Table 6 indicated that in ovine milk fermented by *L. acidophilus*, the addition of hydrolysate (OLA1.5H, OLA3.0H) contributed to an increase in the number of viable bacterial cells at the intestinal phase, compared to the stomach. The opposite occurred in caprine milk, where adding hydrolysate, notably 3%, reduced the number of bacterial cells in the intestine compared to the stomach. Ranadheera et al. [76] also showed that the presence of bile acid salts significantly reduced the viability of probiotics in fermented caprine milk during in vitro gastrointestinal digestion [76]. In a study by Ayyash et al. [77], the reduction in bacterial counts was up to 4.6 logs in fermented bovine milk after the stomach and intestinal digestion phases.

In this study, the *L. rhamnosus* strain was the only strain that regenerated and grew in sheep’s and goat’s milk under small intestinal conditions, compared to the stomach phase (Table 4). Compared to the stomach phase, this strain significantly increased its count in the presence of bovine collagen and hydrolysate only in caprine milk. Moumita et al. [78] found that some bacterial strains become dormant due to acid shock in the stomach and regain their growth when the pH in the small intestine reaches 6.0. A study by Galdino et al. [68] showed the greatest decrease in the number of viable cells after the stomach phase, while an increase in the number of lactic acid bacilli was observed after the last intestinal phase. According to Ziarno and Zaręba [67], bile affects the phospholipids and proteins of the bacterial cell membrane and disrupts macromolecular stability and cellular homeostasis [67].

**Figure 1:**
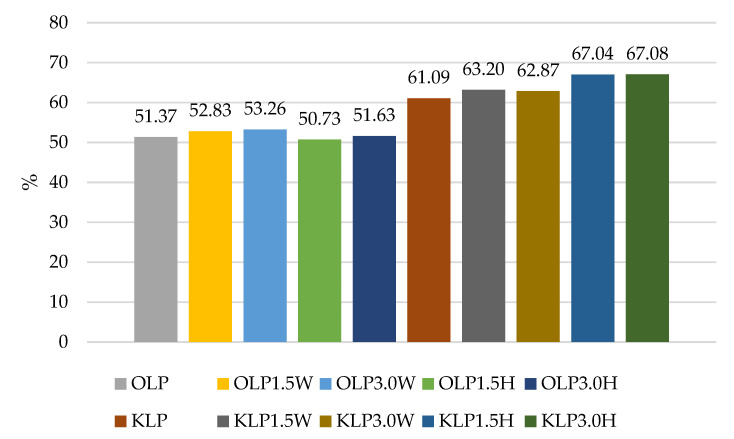
Survival rates (%) in sheep’s and goat’s milk fermented by *Lacticaseibacillus paracasei*.

**Figure 2:**
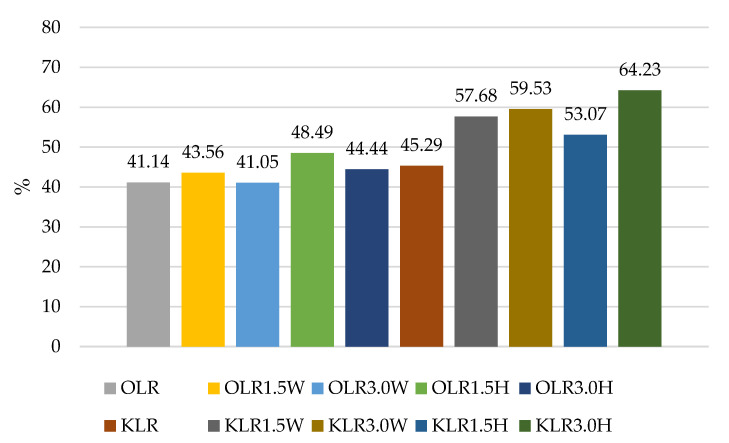
Survival rates (%) in sheep’s and goat’s milk fermented by *Lacticaseibacillus rhamnosus*.

**Figure 3:**
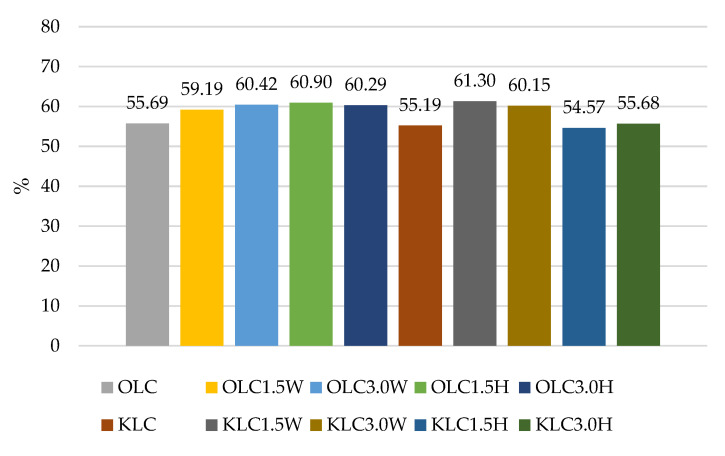
Survival rates (%) in sheep’s and goat’s milk fermented by *Lacticaseibacillus casei*.

**Figure 4:**
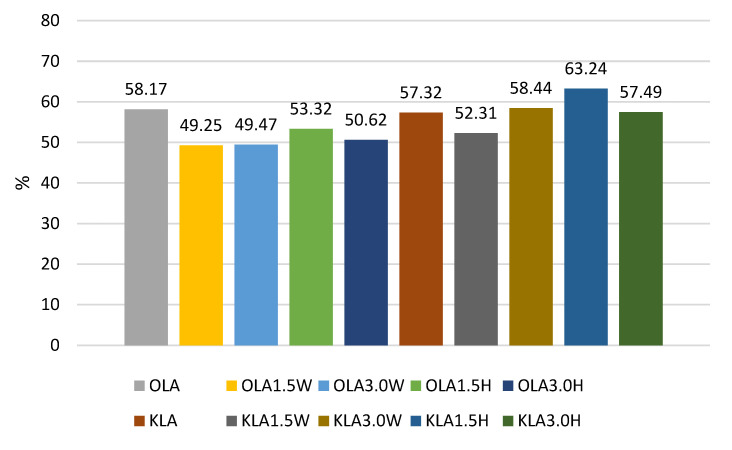
Survival rates (%) in sheep’s and goat’s milk fermented by *Lactobacillus acidophilus*.

The results of our experiment confirmed the literature data for various strains having different survival rates after exposure to a simulated gastrointestinal environment in vitro, as shown in Figure 1, Figure 2, Figure 3 and Figure 4. Some strains survive at high rates, while others do not. In this study, two strains had the most favorable survival under simulated in vitro digestion conditions compared to the number of cells before digestion, *L. casei* and *L.paracasei*, and the survival rates were higher than 50% in each batch (Figure 1 and Figure 3). Radicioni et al. [79] and Ferrario et al. [80] found that differences in the composition of the bacterial envelope and the ability to produce exopolysaccharides could account for the better survival rates of *L. paracasei* and *L. casei*. Exopolysaccharides (EPSs), similarly to fat globules, surround bacterial cells and protect them from harmful conditions and from cell dehydration, antibiotics, toxic substances, osmotic stress, and pathogens [16,81]. Balzaretti et al. [82] found that the *L. paracasei* strain synthesizes a rhamnose-rich bacterial-surface-associated hetero-exopolysaccharide composed of L-rhamnose, D-galactose, and N-acetyl-d-galactosamine in a ratio of 4:1:1. Other strains also produce exopolysaccharides but in varying quantities and structures. 

The survival rates of the *L. acidophilus* strain ranged from 49.25% to 63.24% (Figure 4). In this case, the addition of collagen and hydrolysate to ovine milk adversely affected the survival rate of this probiotic. In caprine milk, a better survival rate was found in milk with 1.5% KLA1.5H hydrolysate than control KLA, by about 6%. In contrast, the survival rate of *L. rhamnosus* was differentiated by the type of milk, and in caprine milk it ranged from 45.29% in KLR to 64.23% in KLR3.0H, while in ovine milk from 41.05% in OLR3.0W to 48.49% in OLR1.5H (Figure 2). Caprine milk also showed better survival of *L. paracasei*, especially with the addition of hydrolysate. Compared to KLP control goat’s milk, adding 3% hydrolysate increased the survival of *L. paracasei* by about 6% in KLP3.0H. In sheep’s milk, adding 3% collagen was more effective, increasing the survival of this strain by 2% in OLP3.0W (Figure 1).

The survival rate of the *L. rhamnosus* strain in OLR fermented control ovine milk was only 41.14%, while in KLR caprine milk it was 45.29%. Similarly, in a study by Leeuwendaal et al. [83], milk fermented by *L. rhamnosus* had the highest reduction in the number of viable strains [83]. In our study, the survival rate of this strain was favorably affected by the addition of bovine collagen, especially in caprine milk, by increasing the survival rate by about 14% compared to the control sample. The addition of 3% hydrolysate to ovine and caprine milk increased the survival of the *L. rhamnosus* strain by 3% and 19%, respectively (Figure 2).

According to Ziarno and Zaręba [67], bacterial survival depends on the initial number of bacterial cells. The higher the number of inserted microorganisms, the more bacterial cells can survive in the intestinal juice [67]. Our study did not confirm this thesis because, before digestion, ovine milk fermented by *L. rhamnosus* had a viable cell count above 10 log cfu g^−1^, and the survival rate was only 41.05–48.49% (Figure 2). Although ovine milk fermented by *L. casei* had a count of 9.5–9.7 log cfu g^−1^ before digestion, the survival rate was higher, ranging from 55.69% to 60.90% (Figure 3). According to Moumit et al. [78], one explanation for the survival and good concentration of microorganisms in fermented milk is that the fat globules may have a protective effect on probiotic cells, and that the presence of milk proteins, mainly casein, is associated with this protection [78]. The lipid fraction is involved in the biosynthesis of fatty acids concentrated around the probiotic plasma membrane [84,85]. Milk proteins have a better buffering capacity to protect cells from difficult environmental conditions [78]. The buffering capacity of dairy products has a protective effect on probiotic bacteria exposed to digestion [68]. According to Ranadheera et al. [31], higher fat and protein levels in ovine milk may protect probiotics passing through the digestive tract. However, this thesis was not confirmed by our study, as there was better survival of bacteria in goat’s milk than in sheep’s milk. Generally, it is known that ovine milk has a higher fat and protein content, including casein, than caprine milk.

An ANOVA analysis of variance indicated that the survival of probiotic bacteria cells was influenced by the strain of bacteria (*p* = 0.0001), type of milk (*p* = 0.0000), and type of collagen (*p* = 0.0120), as well as the interaction of these three factors (*p* = 0.0000). However, the dose of collagen and hydrolysate (1.5% and 3.0%) did not significantly affect bacterial cell survival.

## 4. Conclusions

The conducted studies indicated that fermented caprine and ovine milk could be a suitable matrix for providing probiotics from commercial dairy cultures and contribute to intestinal homeostasis. The results showed that milk fermented by *L. casei* and *L. paracasei* in the small intestine phase showed a higher probiotic content, exceeding 5 log cfu g^−1^, and demonstrated a survival rate above 50%. In contrast, *L. rhamnosus* and *L. acidophilus* strains were identified at cell counts exceeding 4 log cfu g^−1^. In this study, poor survival in simulated in vitro digestion conditions compared to pre-digestion cell counts was observed for *L. rhamnosus*, which showed the most significant reduction in viable cell counts. The type of milk and collagen influenced the survival rate of probiotic bacteria cells. The addition of collagen and hydrolysate to ovine milk resulted in a reduction in the viability of *L. acidophilus*. In contrast, introducing hydrolysate into caprine milk increased the survival of this strain. Adding bovine collagen favorably increased the survival of *L. casei* and *L. rhamnosus* under simulated in vitro digestion conditions.

Evaluating the effects of the addition of bovine collagen and hydrolysate on the survival of probiotic bacterial strains in the gastrointestinal tract would be beneficial for developing new, innovative products with improved health properties and customized for the needs of the intestinal ecosystem.

## Figures and Tables

**Table 1:** Fermented sheep’s milk batches obtained in the experiment [48].

Probiotic Strain	Batch				
	Control	1.5% Bovine Collagen	3.0% Bovine Collagen	1.5% Collagen Protein Hydrolysate	3.0% Collagen Protein Hydrolysate
*Lacticaseibacillus casei* 431	OLC	OLC1.5W	OLC3.0W	OLC1.5H	OLC3.0H
*Lacticaseibacillus paracasei* L-26	OLP	OLP1.5W	OLP3.0W	OLP1.5H	OLP3.0H
*Lactobacillus acidophilus* LA-5	OLA	OLA1.5W	OLA3.0W	OLA1.5H	OLA3.0H
*Lacticaseibacillus rhamnosus* Lr-32	OLR	OLR1.5W	OLR3.0W	OLR1.5H	OLR3.0H

**Table 2:** Fermented goat’s milk batches obtained in the experiment [49].

Probiotic Strain	Batch				
	Control	1.5% Bovine Collagen	3.0% Bovine Collagen	1.5% Collagen Protein Hydrolysate	3.0% Collagen Protein Hydrolysate
*Lacticaseibacillus casei* 431	KLC	KLC1.5W	KLC3.0W	KLC1.5H	KLC3.0H
*Lacticaseibacillus paracasei* L-26	KLP	KLP1.5W	KLP3.0W	KLP1.5H	KLP3.0H
*Lactobacillus acidophilus* LA-5	KLA	KLA1.5W	KLA3.0W	KLA1.5H	KLA3.0H
*Lacticaseibacillus rhamnosus* Lr-32	KLR	KLR1.5W	KLR3.0W	KLR1.5H	KLR3.0H

**Table 3:** Viable counts of bacteria (log cfu g^−1^) in sheep’s and goat’s milk fermented by *Lacticaseibacillus paracasei* before digestion in the oral cavity, stomach, and small intestine.

Experimental Batch	Viable Counts of Probiotic Bacteria, log cfu g^−1^			
	Digestion Phase			
	Before Digestion	Oral Cavity	Stomach	Small Intensine
OLP	10.92 ^Cb^ ± 0.14	10.50 ^Bb^ ± 0.17	5.40 ^Ba^ ± 0.13	5.61 ^Aa^ ± 0.21
OLP1.5W	10.77 ^Cb^ ± 0.15	10.84 ^Cb^ ± 0.12	5.46 ^Ba^ ± 0.14	5.69 ^Aa^ ± 0.11
OLP3.0W	10.89 ^Cb^ ± 0.04	10.85 ^Cb^ ± 0.12	5.52 ^Ba^ ± 0.16	5.80 ^Aa^ ± 0.11
OLP1.5H	10.90 ^Cb^ ± 0.25	10.92 ^Cb^ ± 0.03	5.55 ^Ba^ ± 0.05	5.53 ^Aa^ ± 0.11
OLP3.0H	10.46 ^Bb^ ± 0.23	10.53 ^BCb^ ± 0.37	5.35 ^Ba^ ± 0.14	5.40 ^Aa^ ± 0.12
KLP	9.02 ^Ab^ ± 0.22	9.09 ^Ab^ ± 0.11	5.06 ^Aa^ ± 0.27	5.51 ^Aa^ ± 0.16
KLP1.5W	9.24 ^Ab^ ± 0.18	9.17 ^Ab^ ± 0.39	5.84 ^Ca^ ± 0.05	5.84 ^Ba^ ± 0.09
KLP3.0W	9.40 ^Ab^ ± 0.23	9.45 ^Ab^ ± 0.68	5.92 ^Ca^ ± 0.20	5.91 ^Aa^ ± 0.45
KLP1.5H	8.92 ^Ac^ ± 0.16	8.77 ^Ac^ ± 0.18	5.45 ^Ba^ ± 0.21	5.98 ^Bb^ ± 0.17
KLP3.0H	8.90 ^Ac^ ± 0.22	8.70 ^Ac^ ± 0.30	5.47 ^Ba^ ± 0.19	5.97 ^Bb^ ± 0.07

Mean ± standard deviation; ^a–c^—mean values denoted in rows by different letters differ statistically significantly at *p* ≤ 0.05; ^A–C^—mean values in columns obtained for a single bacterial strain denoted by different letters differ significantly *p* ≤ 0.05.

**Table 4:** Viable counts of bacteria (log cfu g^−1^) in sheep’s and goat’s milk fermented by *Lacticaseibacillus rhamnosus* before digestion in the oral cavity, stomach, and small intestine.

Experimental Batch	Viable Counts of Probiotic Bacteria, log cfu g^−1^			
	Digestion Phase			
	Before Digestion	Oral Cavity	Stomach	Small Intensine
OLR	10.21 ^Cb^ ± 0.23	9.90 ^Ab^ ± 0.24	4.06 ^Ba^ ± 0.08	4.20 ^Aa^ ± 0.40
OLR1.5W	10.17 ^Cb^ ± 0.21	9.88 ^ABb^ ± 0.15	3.58 ^Aa^ ± 0.68	4.43 ^Aa^ ± 0.34
OLR3.0W	10.50 ^Db^ ± 0.24	9.88 ^Bb^ ± 0.42	3.78 ^Aa^ ± 0.62	4.31 ^Aa^ ± 0.39
OLR1.5H	10.27 ^Cb^ ± 0.28	9.79 ^Abb^ ± 0.21	3.65 ^Aa^ ± 0.16	4.98 ^Aa^ ± 0.97
OLR3.0H	10.26 ^Cb^ ± 0.08	9.85 ^Ab^ ± 0.14	3.87 ^Aa^ ± 0.32	4.56 ^Aa^ ± 0.36
KLR	9.23 ^Bb^ ± 0.13	9.25 ^ABb^ ± 0.38	4.02 ^Ba^ ± 0.52	4.18 ^Aa^ ± 0.19
KLR1.5W	9.05 ^Ac^ ± 0.20	8.92 ^Ac^ ± 0.18	3.69 ^Aa^ ± 0.58	5.22 ^Bb^ ± 0.22
KLR3.0W	8.97 ^Ac^ ± 0.07	8.99 ^Ac^ ± 0.29	4.10 ^Ba^ ± 0.13	5.34 ^Bb^ ± 0.21
KLR1.5H	9.12 ^Ac^ ± 0.14	9.07 ^Ac^ ± 0.22	4.00 ^Ba^ ± 0.06	4.84 ^Ab^ ± 0.61
KLR3.0H	9.17 ^ABc^ ± 0.40	8.99 ^Ac^ ± 0.11	4.28 ^Ba^ ± 0.17	5.89 ^Bb^ ± 0.30

Mean ± standard deviation; ^a–c^—mean values denoted in rows by different letters differ statistically significantly at *p* ≤ 0.05; ^A–D^—mean values in columns obtained for a single bacterial strain denoted by different letters differ significantly *p* ≤ 0.05.

**Table 5:** Viable counts of bacteria (log cfu g^−1^) in sheep’s and goat’s milk fermented by *Lacticaseibacillus casei* before digestion in the oral cavity, stomach, and small intestine.

Experimental Batch	Viable Counts of Probiotic Bacteria, log cfu g^−1^			
	Digestion Phase			
	Before Digestion	Oral Cavity	Stomach	Small Intensine
OLC	9.57 ^Bc^ ± 0.34	9.75 ^Cc^ ± 0.09	4.94 ^Ba^ ± 0.11	5.33 ^Bb^ ± 0.19
OLC1.5W	9.68 ^Bb^ ± 0.34	9.74 ^Cb^ ± 0.09	5.72 ^Ca^ ± 0.18	5.73 ^Ca^ ± 0.11
OLC3.0W	9.60 ^Bc^ ± 0.09	9.57 ^Bc^ ± 0.14	5.74 ^Cb^ ± 0.04	5.80 ^Ca^ ± 0.09
OLC1.5H	9.54 ^Bb^ ± 0.04	9.47 ^Bb^ ± 0.10	5.79 ^Ca^ ± 0.14	5.81 ^Ca^ ± 0.28
OLC3.0H	9.52 ^Bb^ ± 0.05	9.42 ^Bb^ ± 0.06	5.78 ^Ca^ ± 0.78	5.74 ^Ca^ ± 0.16
KLC	9.15 ^Ac^ ± 0.39	9.44 ^Bc^ ± 0.12	4.25 ^Aa^ ± 0.06	5.05 ^Ab^ ± 0.21
KLC1.5W	9.56 ^ABc^ ± 0.47	9.38 ^Ac^ ± 0.21	5.65 ^Ca^ ± 0.19	5.86 ^Ba^ ± 0.14
KLC3.0W	9.51 ^Bb^ ± 0.13	9.58 ^Bb^ ± 0.18	5.72 ^Ca^ ± 0.09	5.72 ^Ba^ ± 0.22
KLC1.5H	9.20 ^Ac^ ± 0.12	9.10 ^Ac^ ± 0.28	5.81 ^Cb^ ± 0.14	5.02 ^Aa^ ± 0.37
KLC3.0H	9.07 ^Ab^ ± 0.21	8.95 ^Ab^ ± 0.16	5.64 ^Ca^ ± 0.36	5.05 ^Aa^ ± 0.50

Mean ± standard deviation; ^a–c^—mean values denoted in rows by different letters differ statistically significantly at *p* ≤ 0.05; ^A–C^—mean values in columns obtained for a single bacterial strain denoted by different letters differ significantly *p* ≤ 0.05.

**Table 6:** Viable counts of bacteria (log cfu g^−1^) in sheep’s and goat’s milk fermented by *Lactobacillus acidophilus* before digestion in the oral cavity, stomach, and small intestine.

Experimental Batches	Viable Counts of Probiotic Bacteria, log cfu g^−1^			
	Digestion Phase			
	Before Digestion	Oral Cavity	Stomach	Small Intensine
OLA	9.18 ^ABb^ ± 0.10	9.14 ^Bb^ ± 0.18	4.72 ^Aa^ ± 0.45	5.34 ^Ba^ ± 0.37
OLA1.5W	9.34 ^Bb^ ± 0.06	9.35 ^Cb^ ± 0.11	4.73 ^Aa^ ± 0.49	4.60 ^Aa^ ± 0.39
OLA3.0W	9.38 ^Bb^ ± 0.09	9.31 ^Cb^ ± 0.34	4.42 ^Aa^ ± 0.61	4.64 ^Aa^ ± 0.29
OLA1.5H	9.49 ^Bb^ ± 0.11	9.42 ^Cb^ ± 0.18	4.46 ^Aa^ ± 0.68	5.06 ^Ba^ ± 0.28
OLA3.0H	9.76 ^Bb^ ± 0.11	9.70 ^Cb^ ± 0.18	4.72 ^Aa^ ± 0.28	4.94 ^Ba^ ± 0.20
KLA	8.95 ^Ab^ ± 0.38	8.82 ^Bb^ ± 0.24	5.45 ^Ba^ ± 0.12	5.13 ^Ba^ ± 0.19
KLA1.5W	8.87 ^Ab^ ± 0.11	8.60 ^Ab^ ± 0.25	5.27 ^Ba^ ± 0.22	4.64 ^Aa^ ± 0.60
KLA3.0W	8.71 ^Ab^ ± 0.62	8.36 ^Ab^ ± 0.54	4.85 ^Aa^ ± 0.59	5.09 ^Ba^ ± 0.34
KLA1.5H	8.27 ^Ab^ ± 0.19	8.44 ^Ab^ ± 0.39	5.37 ^Ba^ ± 0.14	5.23 ^Ba^ ± 0.71
KLA3.0H	8.28 ^Ac^ ± 0.11	8.22 ^Ac^ ± 0.27	5.10 ^Bb^ ± 0.05	4.76 ^Aa^ ± 0.77

Mean ± standard deviation; ^a–c^—mean values denoted in rows by different letters differ statistically significantly at *p* ≤ 0.05; ^A–C^—mean values in columns obtained for a single bacterial strain denoted by different letters differ significantly *p* ≤ 0.05.

## Data Availability

The original data presented in the study are included in the article; further inquiries can be directed to the corresponding author.
